# Usefulness of a novel transarterial chemoinfusion plus external‐beam radiation therapy for advanced hepatocellular carcinoma with tumor thrombi in the inferior vena cava and right atrium: Case study

**DOI:** 10.1002/cnr2.1539

**Published:** 2021-08-24

**Authors:** Tomotake Shirono, Hironori Koga, Takashi Niizeki, Hiroaki Nagamatsu, Hideki Iwamoto, Shigeo Shimose, Masahito Nakano, Shusuke Okamura, Yu Noda, Naoki Kamachi, Ryoko Kuromatsu, Etsuyo Ogo, Takuji Torimura

**Affiliations:** ^1^ Division of Gastroenterology, Department of Medicine Kurume University School of Medicine Kurume Japan; ^2^ Department of Gastroenterology Juntendo University School of Medicine Tokyo Japan; ^3^ Department of Radiology Kurume University School of Medicine Kurume Japan

**Keywords:** cardiac invasion, hepatic arterial infusion chemoembolization, oncologic emergency, sorafenib, tumor thrombus

## Abstract

**Background:**

Invasion beyond inferior vena cava (IVC) to right atrium (RA) is a rare complication in patients with advanced hepatocellular carcinoma (HCC), and results in fatal oncologic emergencies, including pulmonary embolism and right heart failure.

**Aim:**

As there is no gold standard treatment for unresectable HCC with tumor thrombi involving IVC and RA, we considered it valuable to assess safety and efficacy of a combination of hepatic arterial infusion chemoembolization (HAIC) therapy and external‐beam radiation therapy (EBRT).

**Methods and results:**

The “New FP” was chosen as the HAIC therapy, in which the enhanced permeation and retention effect was achieved using a cisplatin‐Lipiodol suspension combined with continuous infusion of 5‐fluorouracil (5‐FU). Sixteen patients with HCC with tumor thrombi in IVC, RA, and pulmonary arteries were enrolled. modified response evaluation criteria in solid tumors‐based evaluation of response to the combination treatment was as follows: complete response, 6.2% (1 patient); partial response, 81.3% (13 patients); stable disease, 12.5% (2 patients); progressive disease, 0%. The median overall survival time (MST) was 19.0 months. Notably, MST of patients receiving sequential sorafenib monotherapy (39.0 months) was significantly longer than that of the rest (15.3 months).

**Conclusion:**

The combination of New FP and EBRT is an efficacious treatment option for unresectable HCC involving IVC and RA, complicated with pulmonary embolism. Sequential administration of molecular‐targeted drugs may prolong survival in such patients.

## 1 INTRODUCTION

Vascular invasion is a common complication in patients with advanced‐stage hepatocellular carcinoma (HCC). The presence of tumor thrombi in portal vein (PV) or inferior vena cava (IVC) is common in patients with HCC and has been reported in 44%–84% during autopsy.^1^ However, tumor extension beyond the IVC to the right atrium (RA) is rare, and RA invasion is usually associated with extremely poor prognosis due to oncologic emergencies such as fatal pulmonary embolism and right heart failure.^2^, ^3^ Given that there is no gold standard treatment for aggressive HCC, it is empirically treated with surgery,^4^, ^5^ external‐beam radiation therapy (EBRT),^6^ trans‐arterial chemoembolization (TACE),^7^ and systemic chemotherapy^8^ in selected patients. TACE is a guideline‐recommended treatment procedure for advanced HCC globally, but its therapeutic effect on RA tumor thrombi remains discouraging.^7^ Under such circumstances, a limited number of reports suggest that a challenging combination of TACE with EBRT might potentially improve the therapeutic response in aggressive HCCs.^9^ However, the clinical benefit of this treatment outcome is marginal; therefore, the combination has not been widely accepted. In this regard, it is valuable to assess whether another treatment modality, such as hepatic arterial infusion chemoembolization (HAIC), could complement EBRT better than the existing modalities in the treatment of HCC with tumor thrombi involving IVC and RA.

The high efficacy and safety of our novel HAIC regimen called the New FP has previously been reported,^10^, ^11^ and it demonstrated unprecedented periods of progression‐free survival (PFS) (8.9 months) and overall survival (OS) (27.0 months) in patients with HCC macroscopically invading the PV.^11^ The “New FP” therapy consisting of a cisplatin‐Lipiodol suspension and continuous infusion of 5‐fluorouracil (5‐FU) via a reservoir system exerts an enhanced permeation and retention (EPR) effect,^12^ and its unique combinatorial administration provides robust efficacy on HCC with macroscopic vascular invasion compared with the multi‐tyrosine kinase inhibitor (TKI) sorafenib monotherapy.^13^

In the present study, we retrospectively evaluated the efficacy and safety of the combination of New FP and EBRT in 16 patients with HCC with IVC and RA tumor thrombi. In the combination strategy, the New FP was used to treat intrahepatic HCC lesions and tumor thrombi, and EBRT was exclusively applied to the tumor thrombi in IVC and RA.

## 2 PATIENTS AND METHODS

### 2.1 Criteria for treatments

In this multicenter retrospective cohort study of patients with HCC, the following criteria were set for the use of the New FP therapy: (1) tumor thrombosis invading the PV (portal vein invasion [Vp]2–4), (2) patient age >20 years, (3) estimated life expectancy >3 months, (4) platelet count >50 000/μl and leukocyte count >2000/μl, (5) Child‐Pugh class A or class B, and 6) performance status [Eastern Cooperative Oncology Group (ECOG)] level^14^ of 0–2. The study was conducted in accordance with the Declaration of Helsinki, and the protocol was approved by the ethics review committee of Kurume University (Approval No. 20164). Written informed consent was obtained from each patient before enrollment in the study.

### 2.2 Patients

Between April 2008 to August 2018, 263 patients with advanced HCC received the New FP therapy. Of these, 16 cases (13 males and 3 females) with IVC and RA tumor thrombi underwent a combination treatment of the New FP and EBRT. Patient characteristics are summarized in Table 1. The median age of the patients was 66 years. Liver cirrhosis was present in all patients (Child‐Pugh class A; 8, class B; 7, and class C; 1). Anti‐hepatitis C virus antibodies were detected in four patients, hepatitis B virus surface antigen was detected in six patients, and both tests were negative in six patients. Twelve patients had PV tumor thrombi, and the median maximum diameter of the tumor was 12.8 cm (range 4.4–20.3 cm). All patients had tumor thrombi in pulmonary arteries, but they did not require administration of oxygen. Seven patients were treated with sequential sorafenib therapy after combination treatment with the New FP and EBRT. The mean irradiation dosage for HCC thrombi in the IVC and RA was 42.5 Gy (range 30–60 Gy). All patients were free of uncontrolled ascites and hepatic encephalopathy. The following points were analyzed: OS (months), objective response rates (ORR) evaluated according to the modified Response Evaluation Criteria in Solid Tumors (mRECIST),^15^ adverse effects (AEs), and severe complications including pulmonary embolism.

**TABLE 1:** Baseline characteristics of patients

Factor	Number or median (range)
Age (years)	66 (52–87)
PS 0/1/2	4/10/2
Male/female	13/3
HBV/HCV/non‐B, non‐C	6/4/6
Child‐Pugh A/B/C	8/7/1
BCLC stage C/D	15/1
Tumor size (mm)	128 (44–203)
PVTT 0/2/3/4	4/3/3/6
Total bilirubin (mg/dl)	1 (0.36–1.8)
AST (U/L)	69 (33–328)
ALT (U/L)	35 (10–828)
LDH (U/L)	267 (191–519)
γ‐GTP (U/L)	166 (32–682)
ALP (U/L)	455 (240–3256)
Albumin (g/dl)	3.54 (2.48–4.32)
BUN (mg/dl)	13.7 (10.1–23.7)
Creatinine (mg/dl)	0.78 (0.64–1.1)
CRP (mg/dl)	1.05 (0.1–6.06)
WBC (/μl)	5950 (2400‐10 500)
NLR	2.79 (1.37–9.58)
Platelet (×10^4^)	13.15 (6.1–31.3)
AFP (ng/ml)	3037 (2.9–2 124 991)
DCP (U/ml)	21 346 (22–652 228)
Presence of PE	16
Radiation dose (Gy)	42.5 (30–60)
TKI with/without	7/9

Abbreviations: ALP, alkaline phosphatase; ALT, alanine aminotransferase; AST, aspartate aminotransferase; BUN, blood urea nitrogen; CRP, C‐reactive protein; HBV, hepatitis B virus; HCV, hepatitis C virus; BCLC, Barcelona Clinic Liver Cancer; PVTT, portal vein tumor thrombus; LDH, lactate dehydrogenase; NLR, neutrophil‐to‐lymphocyte ratio; WBC, white blood cell; γ‐GTP, gamma‐glutamyl transpeptidase; AFP, alpha‐fetoprotein; DCP, des‐gamma‐carboxy prothrombin; PE, pulmonary embolism; TKI, tyrosine kinase inhibitor.

### 2.3 Protocol of New FP

The cisplatin‐Lipiodol plus 5‐FU regimen in the New FP comprised a combination of 50 mg cisplatin in 5–10 ml Lipiodol and continuous infusion of 5‐FU (1500 mg/5 days). On day 1 of treatment, cisplatin with Lipiodol was injected through a reservoir catheter followed by 5‐FU (250 mg). Then, 5‐FU (1250 mg) was continuously infused using a balloon pump (SUREFUSER PUMP, Nipro Pharma Corporation, Osaka, Japan) over a period of 5 days. This regimen was administered once a week during the first 2 weeks of admission, and a combination of 20 mg cisplatin with Lipiodol and 5‐FU (500–1250 mg) was infused every 2 weeks at the outpatient department as long as possible. The New FP therapy was discontinued when AEs reached level 2 of the Eastern Cooperative Oncology Group classification with the exception of platelet and leukocyte counts of <30 000/μl and 2000/μl, respectively.

### 2.4 External‐beam radiation therapy

Conventional EBRT was performed using a linear accelerator. Computed tomography (CT) scans of the abdomen were used for three‐dimensional conformal radiation therapy (3D‐CRT) planning. The patients were immobilized in the supine position using thermoplastic casts. The helical CT images were transferred to the treatment planning system (Xio, CMS. Inc, USA). 3D‐CRT was designed to target only the IVC and RA tumor thrombi. The total radiation dose was determined based on the location and burden of each tumor thrombus, hepatic functional reserve of the patient, and the irradiation‐related AEs. 3D‐CRT was administered either concurrently or sequentially with the New FP.

### 2.5 Assessment of response to therapy

The primary efficacy endpoint was objective tumor response, while the secondary endpoint was patient survival. The primary endpoint was assessed at 1–3 months after the initial treatment and every 2 months thereafter. Tumors were measured using dynamic CT or dynamic magnetic resonance imaging (MRI). The response to treatment was classified into four categories according to the mRECIST criteria,^15^ together with the European Association for the Study of the Liver (EASL) amendments that take into account the amount of necrotic tumor^16^: as complete response (CR), representing disappearance of all measurable lesions for more than 4 weeks; partial response (PR), representing a decrease in the sum of the longest diameter by more than 30%, and lack of any new lesion for more than 4 weeks; progressive disease (PD), representing an increase in the sum of the longest diameter by more than 25% or appearance of new lesion(s); and stable disease (SD), representing cases categorized as neither PR nor PD for more than 8 weeks.

### 2.6 Assessment of tolerability

Safety and Adverse Events (AEs) were assessed at each study visit by assessing physical conditions and laboratory examination data. The severity of any toxicity was assessed according to the National Cancer Institute Common Toxicity Criteria (version 5). The presence of seven clinical symptoms and signs commonly noted in patients with advanced HCC (ascites, anorexia, jaundice, local pain, lack of energy, malaise or fatigue, and intratumoral hemorrhage) and complications associated with indwelling catheters (e.g., gastro‐duodenal ulcer, infection, thrombosis, and vascular damage) were also assessed.

### 2.7 Statistical analysis

Baseline data were expressed as median and range values. Survival was confirmed until June 30, 2020. Cumulative survival was calculated using the Kaplan–Meier method and compared using the log‐rank test. Independent factors for survival were assessed using the Cox proportional hazards regression model. Statistical significance was set at *P* < .05. SPSS software version 14.0J (SPSS Inc., Chicago, IL, USA) was used for statistical analysis.

## 3 RESULTS

### 3.1 Representative case of complete response

**FIGURE 1:**
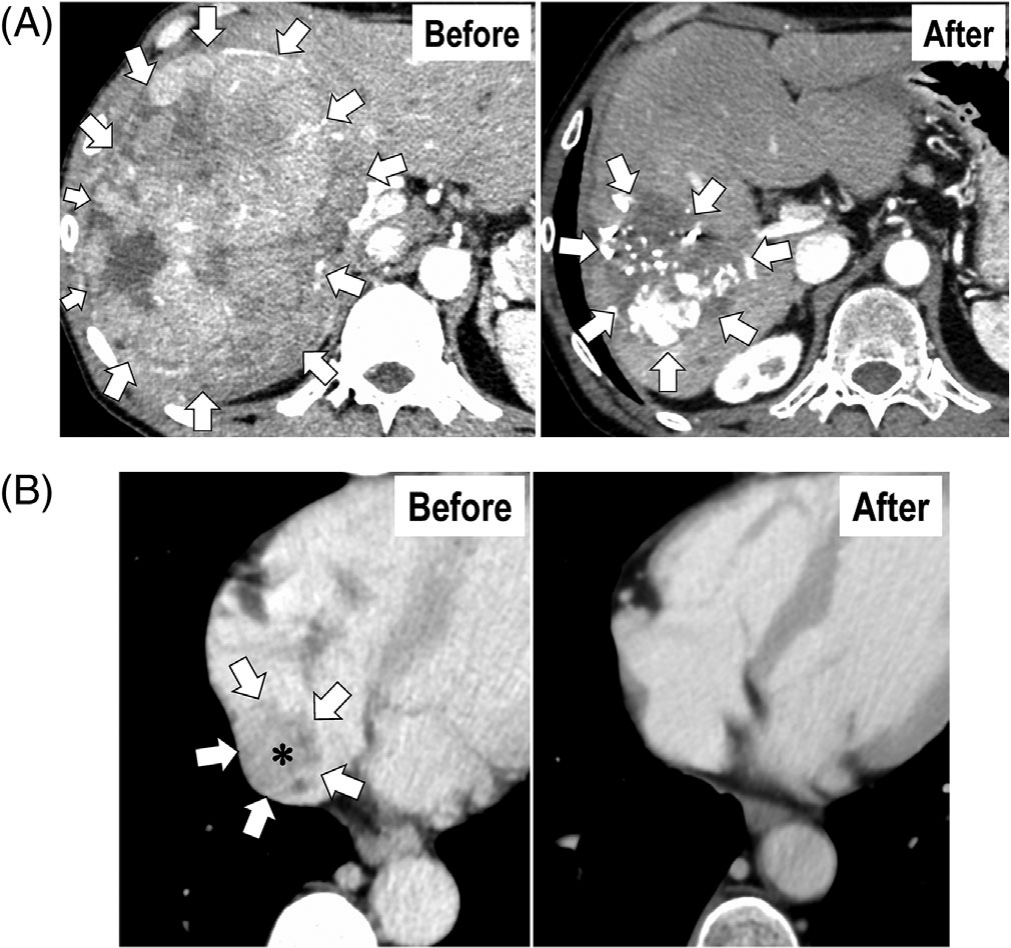
Computed tomography images of a representative patient with unresectable HCC with massive MVI treated with the New FP plus concurrent EBRT. A huge tumor exceeding 20 cm in diameter (A, left image), and massive invasion of the IVC and RA (B, left image) can be seen. White arrows indicate the demarcation of the targeted lesions (A and B). An asterisk (*) demonstrates the tumor thrombus in the RA (B, left image). Following local chemotherapy using the New FP the main tumor in the liver has shrunk remarkably (A, right image), along with the complete disappearance of the tumor thrombus in the RA (B, right image). Before, before the treatment. After, 6 months after the treatment

A patient with huge HCC (≥20.3 cm in length) with Vp4 and hepatic vein invasion (Vv)3 invading the RA was treated with the New FP plus 50 Gy of EBRT. The size of the main tumor in the liver remarkably decreased following local chemotherapy (Figure 1A), along with complete disappearance (CR) of the tumor thrombus in the RA (Figure 1B).

### 3.2 Response rates

**FIGURE 2:**
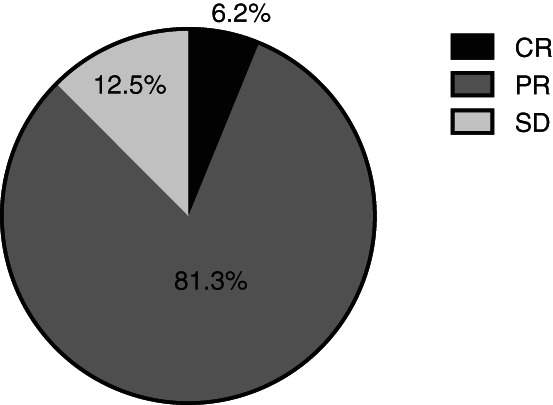
Radiologic assessment of efficacy of the combination treatment based on the mRECIST criteria shows CR 6.2% (1 patient), PR 81.3% (13 patients), SD 12.5% (2 patients), and PD 0%

The results of the mRECIST‐based assessment of the combination of the New FP and EBRT were as follows: CR 6.2% (1 patient), PR 81.3% (13 patients), SD 12.5% (2 patients), PD 0% (Figure 2). The ORR and disease control rates were 87.5% and 100%, respectively.

## 4 OVERALL SURVIVAL

**FIGURE 3:**
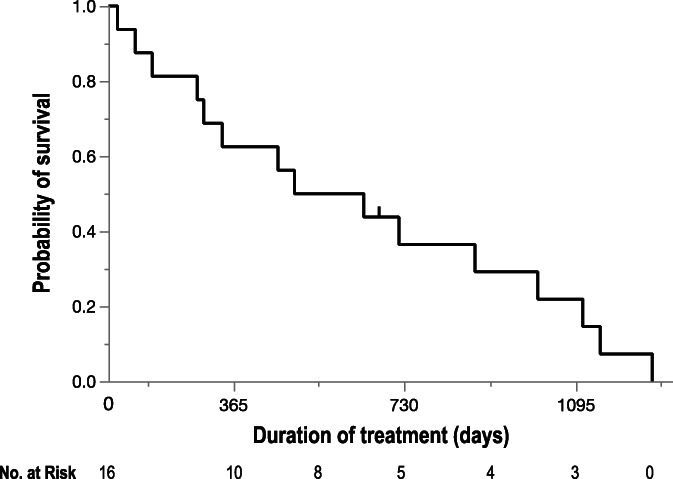
Kaplan–Meier analysis of overall survival in all enrolled patients. The median survival time is 19.0 months. Tick mark indicates censored data

**FIGURE 4:**
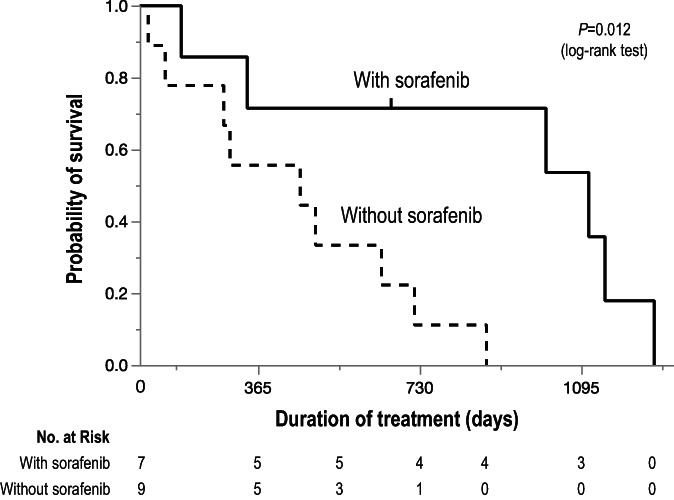
Kaplan–Meier analysis of overall survival in patients with (solid line) and without (dotted line) receiving the multi‐tyrosine kinase inhibitor sorafenib monotherapy after the combination treatment of the New FP and EBRT. MST of the patients sequentially administered sorafenib (39.0 months) is significantly longer than that of the rest (15.3 months) (*P* = .012, log‐rank test). Tick mark indicates censored data

The median overall survival time (MST) of patients enrolled in this study was 19.0 months (Figure 3). Of note, the MST of the patients who sequentially received the multi‐tyrosine kinase inhibitor sorafenib (39.0 months) was significantly longer than that of those not receiving the drug (15.3 months) (*P* = .012, log‐rank test) (Figure 4).

### 4.1 Adverse effects

Grade 3 AEs, such as thrombocytopenia, liver injury, and constrictive pericarditis, were observed in one patient each. Grade 2 esophagitis was found in three patients. Pulmonary embolism was not exacerbated. All AEs resolved with conservative treatment. No specific AEs associated with the combination treatment were reported.

## 5 DISCUSSION

In the current study, we demonstrated the following novel findings in the treatment of highly advanced HCC with massive invasion to the IVC and RA, complicated with pulmonary embolism: (1) high efficacy of the combination of the New FP and EBRT, exhibiting an ORR of 87.5% and an MST of 19.0 months, and (2) a significantly longer survival time in patients receiving sequential sorafenib after the combination treatment.

The high ORR in this study of aggressive HCCs with macrovascular invasion (MVI) could be derived from the mutually complementary cytotoxic effects of the New FP and EBRT. The New FP is known to have a high potential to achieve CR, even when applied to HCCs with tumor thrombi in the PV or IVC,^11^ which is an unsuitable tumor condition or contraindication for TACE. The high ability to control locally advanced HCC is based on the EPR effect,^12^ in which high‐molecular weight non‐targeted drugs and prodrugs accumulate in tissues that offer increased vascular permeability, such as in sites of inflammation or cancer.^12^ In contrast to the strong effect of the New FP on intrahepatic HCC, the therapy cannot completely kill cells within large tumor thrombi in the IVC or RA because of the narrow and fragile vasculature of the sites. In this regard, EBRT is a powerful partner when sequentially or concurrently applied to extrahepatic tumor thrombi invading the IVC or RA. Thus, the total effect of the combination treatment yielded a high ORR in our study.

Curative resection is another therapeutic option for HCCs with massive invasion to the IVC or RA without pulmonary embolism.^4^, ^5^ In a limited number of cohorts, the MST of patients with HCC undergoing curative resection was 19.0–30.8 months,^4^, ^5^ which was significantly longer than that of patients treated with TACE (4.5 months).^4^ In our study, the MST was 19.0 months in patients with inoperable HCC with invasion beyond the IVC to the RA and complicated with pulmonary embolism. Although there is no direct comparison with surgical resection, the combination treatment of the New FP and EBRT had clinical advantages over surgery, including relatively minimum invasiveness and a much longer survival time (39.0 months) if the patients were administered sorafenib sequentially after the combination treatment. In addition, the occurrence of routine manageable AEs was another advantage for combination treatment.

In the era of molecular‐targeted drugs for the treatment of HCC, we should consider the standpoint of the combination treatment of the New FP and EBRT. Recently, the novel treatment regimen “atezolizumab plus bevacizumab” was approved as the first immuno‐oncology therapy for unresectable HCC. In the setting of its phase 1b clinical trial (GO30140), the regimen exhibited an ORR of 35.5% (CR 11.5% and PR 24%, evaluated by RECIST v1.1), although 53% of the enrolled patients with HCC had MVI.^17^ Thus, immuno‐oncology therapy may be promising in controlling far‐advanced HCC with extrahepatic tumor thrombi invading the IVC or RA, as in our patients. However, to date, there is no definite biomarker to predict the responsiveness or time to response to novel treatments. Therefore, such immuno‐oncology therapy should be applied after efficacious and manageable treatments, such as the combination of the New FP and EBRT, in patients under a state of pre‐oncologic emergency with unresectable HCC invading the IVC, RA, and pulmonary arteries. Although the current study was retrospective, and the number of patients was small, the combination of the New FP and EBRT would be feasible in cases where oncologic emergencies must be treated using speedy and effective procedures.

## AUTHOR CONTRIBUTIONS

**Tomotake Shirono:** Conceptualization; data curation; formal analysis; investigation; methodology; writing ‐ original draft. **Hironori Koga:** Conceptualization; data curation; formal analysis; methodology; supervision; writing ‐ original draft; writing‐review & editing. **Takashi Niizeki:** Data curation; writing‐review & editing. **Hiroaki Nagamatsu:** Data curation; writing‐review & editing. **Hideki Iwamoto:** Data curation; writing‐review & editing. **Shigeo Shimose:** Data curation; writing‐review & editing. **Masahito Nakano:** Data curation; writing‐review & editing. **Shusuke Okamura:** Data curation; writing‐review & editing. **Yu Noda:** Data curation; writing‐review & editing. **Naoki Kamachi:** Data curation; writing‐review & editing. **Ryoko Kuromatsu:** Data curation; writing‐review & editing. **Etsuyo Ogo:** Methodology; writing‐review & editing. **Takuji Torimura:** Supervision; writing‐review & editing.

## CONFLICT OF INTEREST

The authors have stated explicitly that there are no conflicts of interest in connection with this article.

## ETHICAL STATEMENT

The study was conducted in accordance with the Declaration of Helsinki, and the protocol was approved by the ethics review committee of Kurume University (Approval No. 20164). Written informed consent was obtained from each patient before enrollment in the study.

## Data Availability

Clinical data are stored in an institutional database and will be shared upon request to the corresponding author.
